# Supplementary material for: Gadd45α modulates aversive learning through post‐transcriptional regulation of memory‐related mRNAs
Source: EMBO Rep. 2019 Apr 4;20(6):e46022. doi: 10.15252/embr.201846022 (PMC6549022; doi:10.15252/embr.201846022)
Supplement: Supplementary file 1 — Appendix [file EMBR-20-e46022-s001.pdf]

Gadd45α modulates aversive learning through post-transcriptional regulation of memory-related mRNAs

Alejandro Aparisi Rey[1], Emil Karaulanov[2], Salim Sharopov[3], Khelifa Arab[2], Andrea Schäfer[2], Mathias Gierl[2], Stephan Guggenhuber[1], Caroline Brandes[1], Luigi Pennella[1], Wolfram H Gruhn[2], Ruth Jelinek[1], Christina Maul[1], Andrea Conrad[1], Werner Kilb[3], Heiko J Luhmann[3], Christof Niehrs[2,4,*] & Beat Lutz[1,**]

1 Institute of Physiological Chemistry, University Medical Center of the Johannes Gutenberg University Mainz, Mainz, Germany
2 Institute of Molecular Biology, Mainz, Germany
3 Institute of Physiology, University Medical Center of the Johannes Gutenberg University Mainz, Mainz, Germany
4 Division of Molecular Embryology, DKFZ-ZMBH Alliance, Deutsches Krebsforschungszentrum (DKFZ), Heidelberg, Germany

* Corresponding author. Tel: +49 6131 39 21400; E-mail: c.niehrs@imb-mainz.de
** Corresponding author. Tel: +49 6131 39 25912; E-mail: beat.lutz@uni-mainz.de

Appendix - Table of contents

Page 2 Appendix Figure S1. Principal component analysis and metagene profiles of RNA-seq transcript coverage.

Page 3 Appendix Figure S2. UCSC browser views of *Grin2a* in all the samples included in the RNA-seq experiment.

Page 4 Appendix Figure S3. UCSC browser views of Gadd45α affected transcripts

Page 5 Appendix Figure S4. Scheme of the RNA fragmentation of a given Gadd45α target gene and the detection outcome depending on the priming strategy during reverse-transcription.

Page 6 Appendix Figure S5. qPCR validation of Gadd45α-regulated genes in two independent sets of samples

Page 7 Appendix Figure S6. Detailed analysis of exon usage and qPCR validation assays

Page 8 Appendix Table S1. Detailed exonic read coverage of Gadd45α-target genes

Page 9 Appendix Table S2. List of primers

Appendix Figure S1

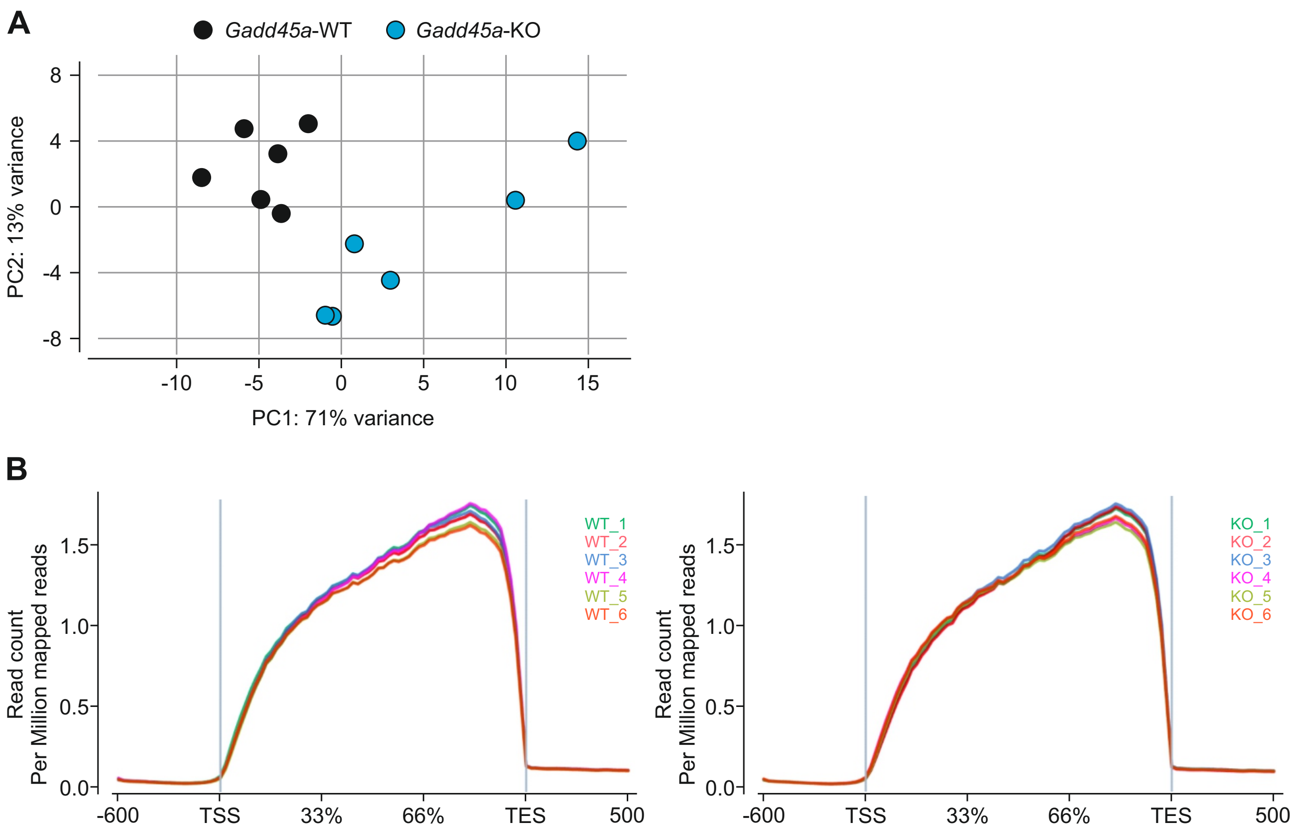

Appendix Figure S1. Principal component analysis and metagene profiles of RNA-seq transcript coverage.

A Principal component analysis plot of sample similarity based on variance stabilization transformation-normalised read counts per gene of *Gadd45a*-WT (black, n=6) and *Gadd45a*–KO samples (blue, n=6).

B Average read density profiles summarised over all transcripts between their transcription start site (TSS) and transcription end site (TES) revealed a similar bias towards the mRNA 3´ end in both *Gadd45a*-WT (left, n=6) and *Gadd45a*–KO samples (right, n=6), explainable by the polyA selection procedure during NGS library preparation.

Appendix Figure S2

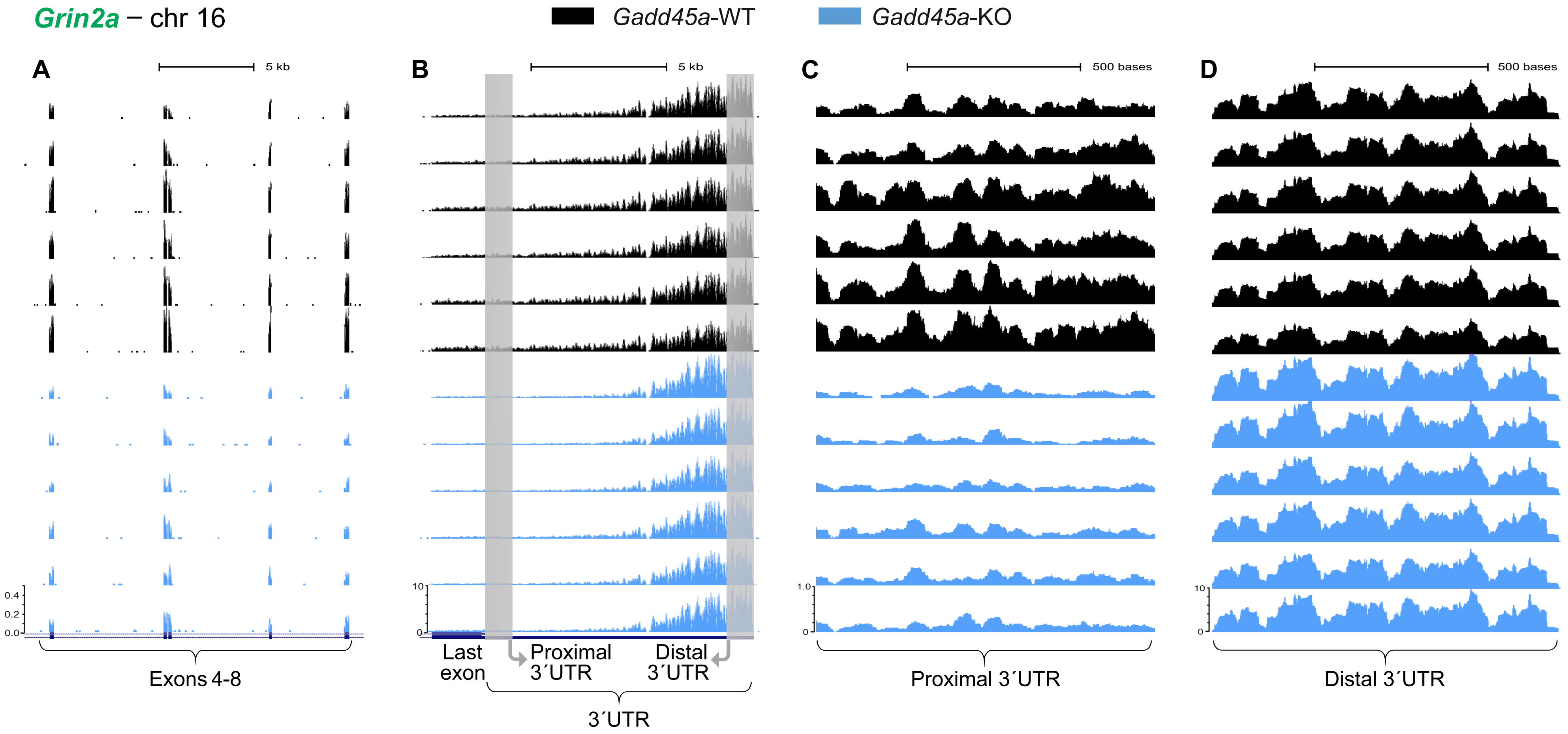

Appendix Figure S2. UCSC browser views of *Grin2a* in all the samples included in the RNA-seq experiment. Note that the read coverage in all regions of the gene is highly reproducible between the 6 replicates of each genotype. The y axes represent reads-per-million normalised RNA-seq coverage (depicted in the lower *Gadd45a*-KO sample). It varies depending on the region, but it is always the same for all samples within one region.

A Read coverage for exons 4-8 is clearly reduced in all *Gadd45a*-KO samples (blue), as compared to *Gadd45a*-WT samples (black).

B Read coverage for the extended 3´UTR of *Grin2a* shows a clear 5´<3´ gradient, with a consistently enhanced steepness in all *Gadd45a*-KO samples.

C Read coverage for the proximal part of the extended 3´UTR of *Grin2a*. Note that the read coverage is significantly lower in all *Gadd45a*-KO samples.

D Read coverage for the distal part of the extended 3´UTR of *Grin2a*. Note that the read coverage is very similar in all samples, independently of the genotype.

Appendix Figure S3

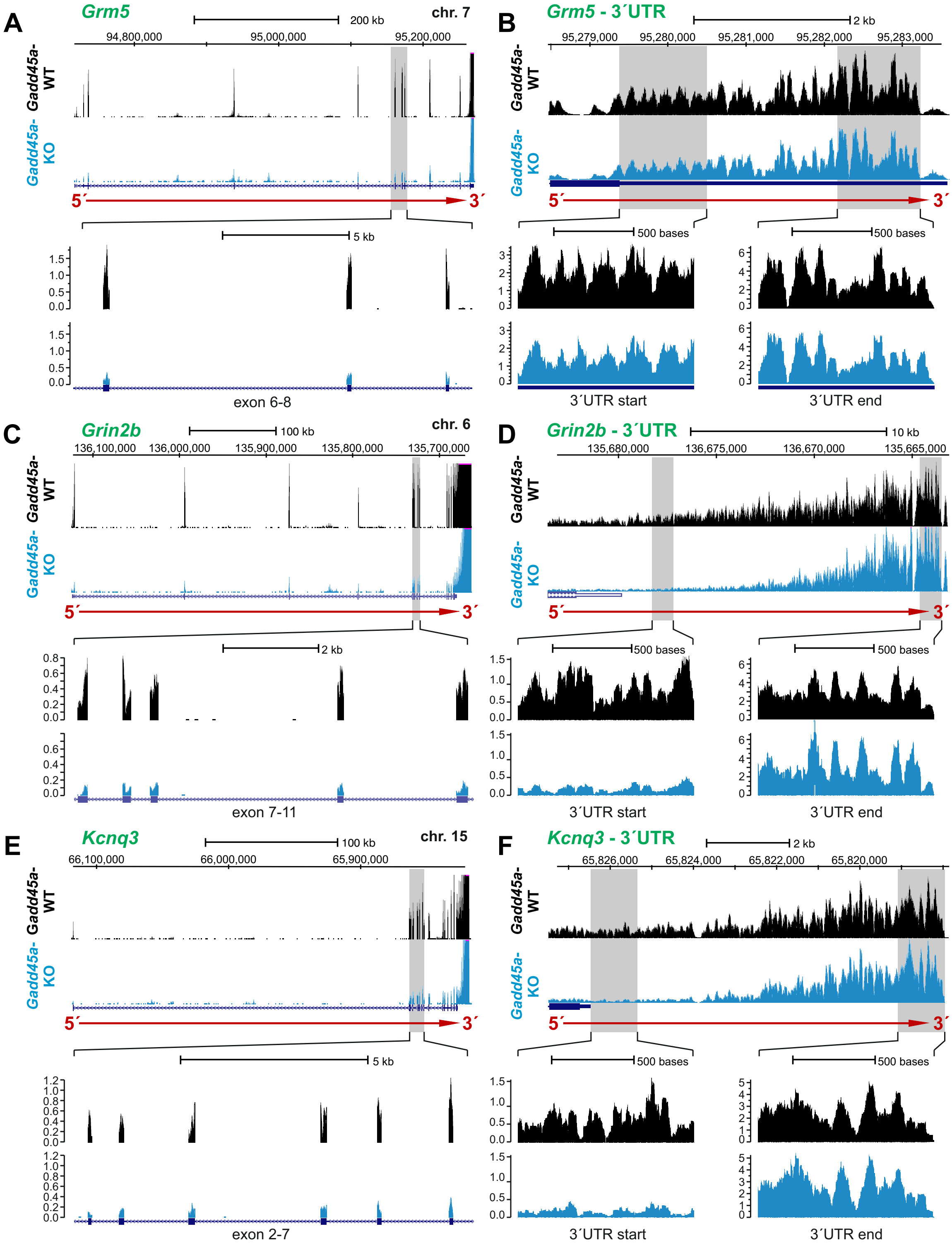

Appendix Figure S3. UCSC browser views of Gadd45α-regulated transcripts. RNA-seq performed on *Gadd45a*-WT (black, n=6) and *Gadd45a*-KO hippocampi (blue, n=6) revealed a number of de-regulated transcripts in *Gadd45a*-KO samples as compared to WT, which contain extended 3´UTRs. For all transcripts, the annotated gene structure (purple line) and the direction of transcription (red arrow) is depicted. Parts of the genes (grey rectangles) are magnified for detail and the y axes represent reads-per-million normalised RNA-seq coverage. Note that for most transcripts reduction in mRNA levels was observed in the gene body (exons) and at the beginning of the 3´UTR, but it was absent at the actual end of the extended 3´UTR, suggesting differential RNA integrity.

A-B Read coverage for the entire *Grm5* transcript (A) with detailed view of its 3´UTR (B).

C-D Read coverage for the entire *Grin2b* transcript (C) with detailed view of its 3´UTR (D).

E-F Read coverage for the entire *Kcnq3* transcript (E) with detailed view of its 3´UTR (F).

Appendix Figure S4

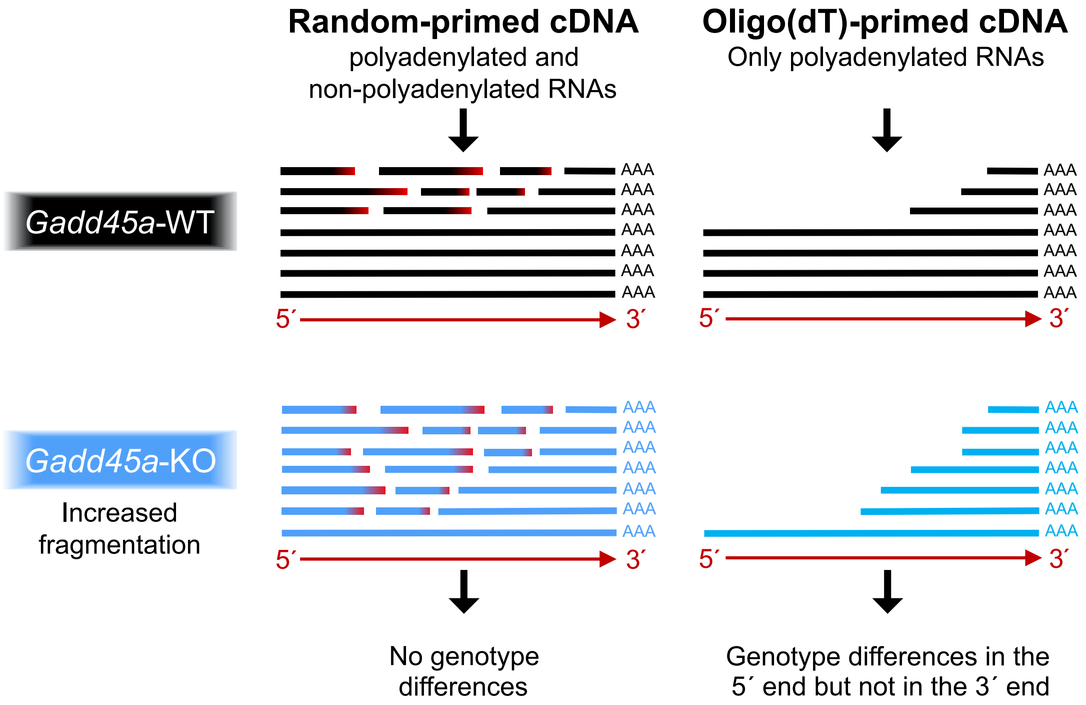

Appendix Figure S4. Scheme of the RNA fragmentation of a given Gadd45α target gene and the detection outcome depending on the priming strategy during reverse-transcription. Note that fragmented RNAs without poly(A) tail (represented by lines with red ends) are measured when using random primers, complicating the detection of RNA stability-dependent effects. Meanwhile, the use of oligo(dT) primers reveals different patterns of expression that are controlled by RNA stabilization.

Appendix Figure S5

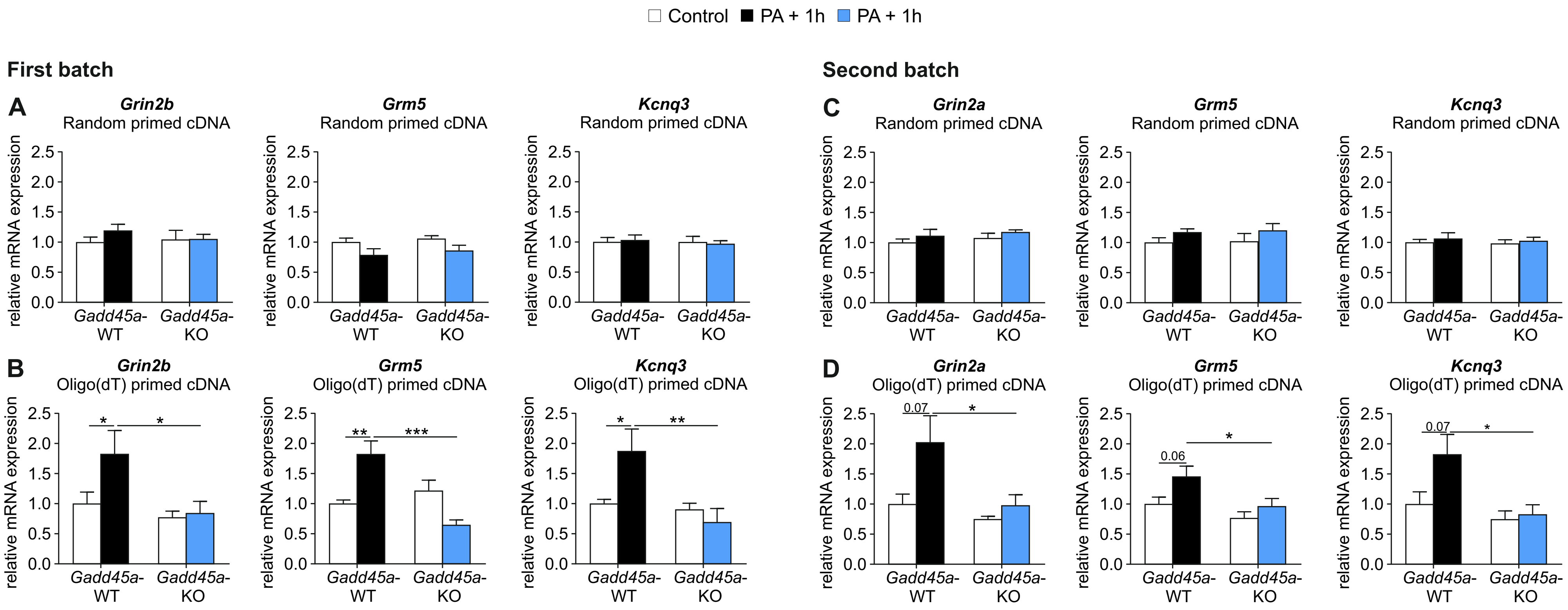

Appendix Figure S5. qPCR validation of Gadd45α-regulated genes in two independent sets of samples

A mRNA levels of *Grin2b*, *Grm5*, and *Kcnq3*. qPCR was performed with random-primed cDNA (analyzing total RNA) obtained from the same batch of samples originally analyzed by RNA-seq. Note that none of the mRNA levels are significantly altered. Values shown are mean ± SEM; 2-Way ANOVA and Bonferroni Post-hoc test.

B mRNA levels of *Grin2b*, *Grm5*, and *Kcnq3*. qPCR was performed with oligo(dT)-primed cDNA (analyzing polyadenylated RNA) obtained from the same batch of samples originally analyzed by RNA-seq. Note that for all the transcripts a significant induction 1h after PA can only be observed in *Gadd45a*-WT mice. Values shown are mean ± SEM; 2-Way ANOVA and Bonferroni Post-hoc test: * = p<0.05, ** = p<0.01, *** = p<0.001.

C mRNA levels of *Grin2a*, *Grm5*, and *Kcnq3*. qPCR was performed with random-primed cDNA (analyzing total RNA) obtained from an independent set of samples (n= 6 for all experimental groups). Note that none of the mRNA levels are significantly altered. Values shown are mean ± SEM; 2-Way ANOVA and Bonferroni Post-hoc test.

D mRNA levels of *Grin2a*, *Grm5*, and *Kcnq3.* qPCR was performed with oligo(dT)-primed cDNA (analyzing polyadenylated RNA) obtained from the same set of samples as in (C). Note that for all the transcripts a significant difference between *Gadd45a*-WT and –KO mice is detected only in PA+1h groups. Values shown are mean ± SEM; 2-Way ANOVA and Bonferroni Post-hoc test: * = p<0.05.

Appendix Figure S6

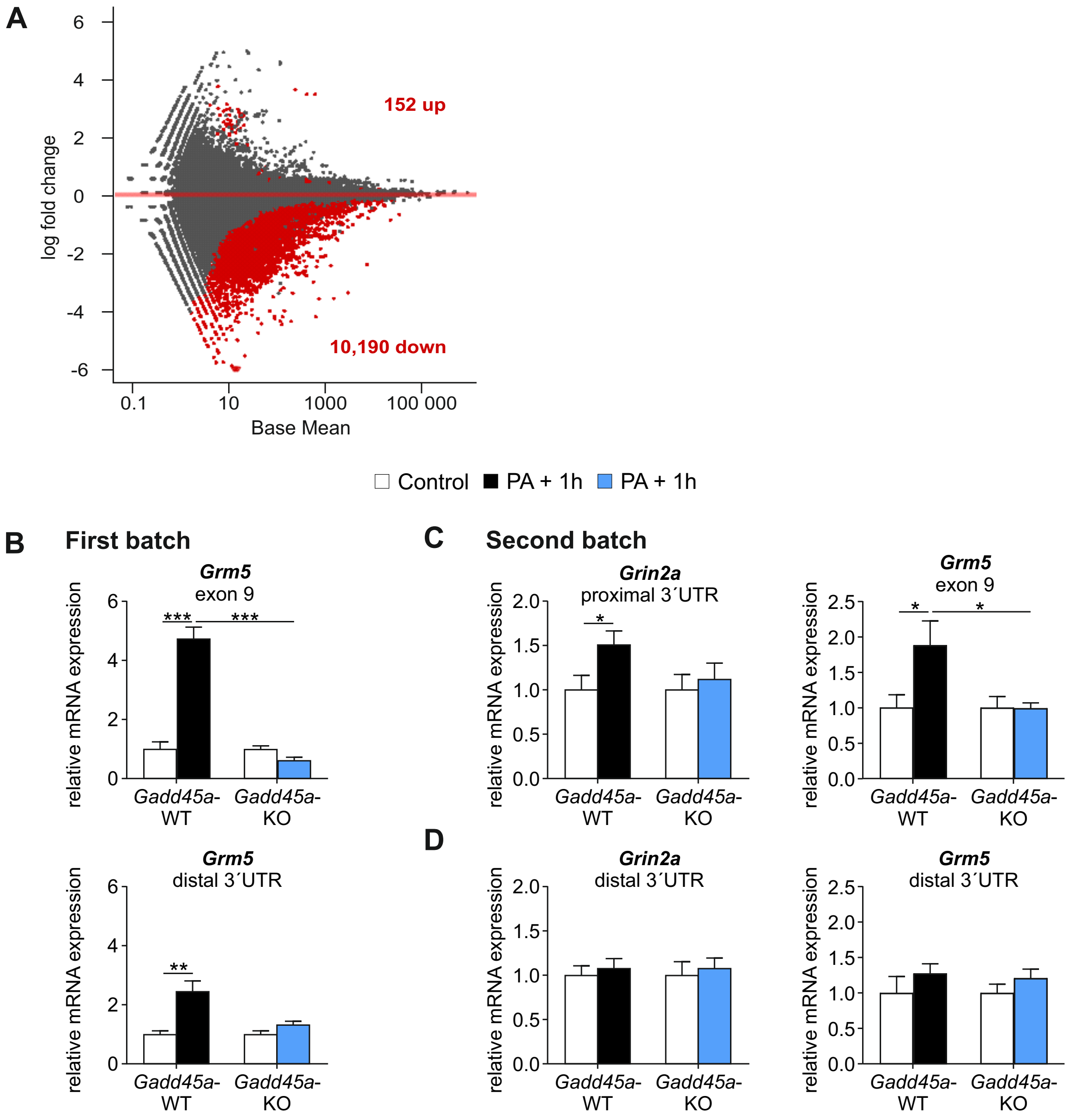

Appendix Figure S6. Detailed analysis of exon usage, and qPCR validation of differential effects on proximal and distal parts of the 3´UTR of Gadd45α-regulated transcripts.

A Differential expression analysis at the level of exons. *Gadd45a*-KO vs. *Gadd45a*-WT differential MA plot of all analysed gene exons (n=357,098) with significant changes (FDR < 0.01) highlighted in red color. Almost all (99%) of the affected exons show down-regulation in *Gadd45a*-KO mice. Most exons of the four genes of interest (*Grin2a, Grin2b, Kcnq3, Grm5*) are also among the differential hits (see details in Appendix Table S1).

B mRNA levels of distal parts of the 3´UTR of *Grm5* compared to that of exon 9 (close to the 3´UTR start) in the same set of samples analyzed in Figure 4. Increased mRNA levels of *Grm5* exon 9 was found only in *Gadd45a*-WT (upper panel). This pattern was similar when analyzing the distal part of the 3´UTR (lower panel), although the induction in *Gadd45a*-WT was less pronounced than that of exon 9. Values shown are mean ± SEM; 2-Way ANOVA and Bonferroni post-hoc test: ** = $p<0.01$, *** = $p<0.001$.

C-D mRNA levels of distal and proximal parts of the 3´UTR of *Grin2a* and *Grm5* (exon 9 instead of proximal 3´UTR) in the same set of samples analyzed in Appendix Figure 5C-D (independent set of samples). Note that in both transcripts, proximal parts of the 3´UTR (exon 9 in the case of *Grm5*) (C) are induced in *Gadd45a*-WT but not in *Gadd45a*-KO; while distal parts (D) remain unchanged. Values shown are mean ± SEM; 2-Way ANOVA and Bonferroni post-hoc test: * = $p<0.05$.

Appendix Table S1.

Detailed exonic read coverage of Gadd45α-target genes

Gene	Analysed Exons (from 5' to 3') with mm9 coordinates	Base Mean	log2. Fold Change	Padj (FDR)
Grin2a	ENSMUSG00000059003:chr16:9994916-9995014	9.021	-1.385	4.44E-02
	ENSMUSG00000059003:chr16:9994152-9994353	11.257	-1.715	2.12E-04
	ENSMUSG00000059003:chr16:9994152-9994342	11.167	-1.701	2.97E-04
	ENSMUSG00000059003:chr16:9992213-9992644	56.716	-1.315	2.02E-08
	ENSMUSG00000059003:chr16:9992213-9992626	56.06	-1.323	1.18E-08
	ENSMUSG00000059003:chr16:9991748-9992644	57.353	-1.327	1.89E-08
	ENSMUSG00000059003:chr16:9761484-9762076	169.606	-1.29	1.45E-05
	ENSMUSG00000059003:chr16:9707697-9707811	35.802	-1.238	4.31E-07
	ENSMUSG00000059003:chr16:9669798-9670003	68.365	-1.302	1.01E-08
	ENSMUSG00000059003:chr16:9663532-9663700	70.801	-1.168	1.73E-04
	ENSMUSG00000059003:chr16:9663274-9663427	46.232	-0.944	5.22E-02
	ENSMUSG00000059003:chr16:9657767-9657892	46.101	-1.318	4.83E-08
	ENSMUSG00000059003:chr16:9653489-9653718	73.799	-1.281	5.56E-08
	ENSMUSG00000059003:chr16:9644192-9644352	63.074	-1.475	9.39E-09
	ENSMUSG00000059003:chr16:9612663-9612850	64.359	-1.514	1.19E-08
	ENSMUSG00000059003:chr16:9585119-9585357	89.426	-1.377	1.58E-09
	ENSMUSG00000059003:chr16:9577920-9579719	639.82	-1.416	6.32E-09
	ENSMUSG00000059003:chr16:9577803-9579719	673.073	-1.405	2.83E-09
	ENSMUSG00000059003:chr16 distalUTR:9567990-9568990 (*)	***6549.175***	***0.232***	***2.29E-02***
Grin2b	ENSMUSG00000030209:chr6:136122883-136123121	21.449	-1.623	4.53E-03
	ENSMUSG00000030209:chr6:136122883-136122955	15.234	-1.624	2.44E-02
	ENSMUSG00000030209:chr6:136121434-136121900	143.474	-1.508	7.48E-03
	ENSMUSG00000030209:chr6:136121434-136121740	110.937	-1.539	8.59E-03
	ENSMUSG00000030209:chr6:135994214-135994337	56.804	-1.328	2.16E-02
	ENSMUSG00000030209:chr6:135993909-135994337	155.162	-1.381	2.04E-02
	ENSMUSG00000030209:chr6:135872890-135873488	217.414	-1.603	4.23E-03
	ENSMUSG00000030209:chr6:135819721-135820408	1.944	0.725	8.61E-01
	ENSMUSG00000030209:chr6:135793106-135793220	43.637	-1.676	7.16E-03
	ENSMUSG00000030209:chr6:135730152-135730354	74.138	-1.58	6.60E-03
	ENSMUSG00000030209:chr6:135729244-135729415	73.689	-1.568	1.67E-02
	ENSMUSG00000030209:chr6:135728679-135728832	73.716	-1.433	2.74E-02
	ENSMUSG00000030209:chr6:135724801-135724926	62.502	-1.596	3.22E-03
	ENSMUSG00000030209:chr6:135722211-135722440	108.591	-1.657	2.31E-03
	ENSMUSG00000030209:chr6:135690940-135691100	76.549	-1.384	7.30E-03
	ENSMUSG00000030209:chr6:135688977-135689164	73.016	-1.678	9.57E-05
	ENSMUSG00000030209:chr6:135686302-135686540	109.313	-1.524	4.06E-03
	ENSMUSG00000030209:chr6:135681645-135683966	866.579	-1.434	3.84E-03
	ENSMUSG00000030209:chr6:135679844-135683966	1531.522	-1.359	3.66E-03
	ENSMUSG00000030209:chr6 - distalUTR:135663253-135664253 (*)	***5064.289***	***0.223***	***1.20E-01***
Kcnq3	ENSMUSG00000056258:chr15:66117398-66117786	29.547	-1.036	6.18E-03
	ENSMUSG00000056258:chr15:65862942-65863032	33.027	-1.199	1.34E-04
	ENSMUSG00000056258:chr15:65862081-65862207	51.845	-1.088	2.11E-04
	ENSMUSG00000056258:chr15:65860187-65860359	80.392	-0.973	2.10E-04
	ENSMUSG00000056258:chr15:65856676-65856831	92.264	-1.126	1.78E-07
	ENSMUSG00000056258:chr15:65855209-65855319	76.113	-1.236	9.60E-08
	ENSMUSG00000056258:chr15:65853303-65853398	75.063	-1.118	3.26E-09
	ENSMUSG00000056258:chr15:65851849-65851943	33.942	-1.261	1.91E-05
	ENSMUSG00000056258:chr15:65848011-65848037	22.815	-0.997	6.83E-03
	ENSMUSG00000056258:chr15:65837334-65837536	96.364	-1.207	2.18E-07
	ENSMUSG00000056258:chr15:65836284-65836386	58.137	-0.949	1.40E-03
	ENSMUSG00000056258:chr15:65833757-65833888	67.581	-1.005	2.98E-05
	ENSMUSG00000056258:chr15:65831579-65831677	71.742	-1.118	5.53E-05
	ENSMUSG00000056258:chr15:65829263-65829347	86.261	-1.209	2.27E-09
	ENSMUSG00000056258:chr15:65826733-65827467	380.005	-1.137	3.39E-11
	ENSMUSG00000056258:chr15 - distalUTR:65817980-65818980 (*)	***3244.558***	***0.103***	***3.79E-01***
Grm5	ENSMUSG00000049583:chr7:94750952-94751711	430.226	-2.022	4.58E-09
	ENSMUSG00000049583:chr7:94751054-94751711	386.062	-1.993	2.56E-09
	ENSMUSG00000049583:chr7:94952325-94952574	190.065	-2.078	5.72E-15
	ENSMUSG00000049583:chr7:94957811-94958130	7.668	-0.331	8.85E-01
	ENSMUSG00000049583:chr7:95123645-95123880	159.395	-2.259	7.08E-12
	ENSMUSG00000049583:chr7:95174934-95175180	165.81	-2.159	8.05E-12
	ENSMUSG00000049583:chr7:95184578-95184746	141.649	-2.128	3.80E-10
	ENSMUSG00000049583:chr7:95188493-95188619	106.935	-2.107	1.79E-29
	ENSMUSG00000049583:chr7:95222701-95223640	755.169	-1.863	1.03E-15
	ENSMUSG00000049583:chr7:95264703-95264798	109.199	-1.69	9.20E-29
	ENSMUSG00000049583:chr7:95278491-95278626	183.309	-1.329	1.13E-21
	ENSMUSG00000049583:chr7:95278491-95279379	655.083	-0.893	9.31E-15
	ENSMUSG00000049583:chr7:95278491-95280164	2294.448	-0.553	6.46E-18
	ENSMUSG00000049583:chr7:95278491-95283417	11763.236	-0.075	6.57E-01
	ENSMUSG00000049583:chr7:95279168-95279404	347.493	-0.629	1.81E-07
	ENSMUSG00000049583:chr7 - distalUTR:95282573-95283573 (*)	***2544.236***	***0.071***	***7.59E-01***

Appendix Table S1. *Gadd45a* -KO vs. *Gadd45a* -WT exonic changes for the four genes of interest in the exon-level differential expression analysis shown in Appendix Fig. S6A. Note that while most canonical exons are significantly down-regulated in *Gadd45a* -KO mice, the distal 1 kb regions of the extended 3'UTRs (*), which were included in the DESeq2 analysis as additional "pseudo-exons", are not significantly affected.

Appendix Table S2.

List of primers

**Taqman assays**			
**Gene symbol**	**Gene name**	**Assay coverage**	**Assay code**
Gusb	Glucoronidase beta	Exons 10-11	Mm01197698_m1
Gadd45a	Growth arrest and DNA-damage inducible protein 45 alpha	Exons 3-4	Mm00432802_m1
Gadd45b	Growth arrest and DNA-damage inducible protein 45 beta	Exons 1-2	Mm00435121_g1
Gadd45g	Growth arrest and DNA-damage inducible protein 45 gamma	Exons 2-3	Mm01352550_g1
Arc	Activity regulated cytoskeletal-associated protein	Exons 2-3	Mm01204954_g1
Grin2a	Glutamate receptor, ionotropic, NMDA2A (epsilon 1)	Exons 3-4	Mm00433802_m1
Grin2b	Glutamate receptor, ionotropic, NMDA2B (epsilon 2)	Exons 13-14	Mm00433820_m1
Kcnq3	Potassium voltage-gated channel, subfamily Q, member 3	Exons 4-5	Mm00548884_m1
Reln	Reelin	Exons 22-23	Mm00465200_m1
Bdnf	brain derived neurotrophic factor	Exon 4	Mm00432069_m1
Grm5	Metabotropic glutamate receptor 5	Exon 4	Mm00690332_m1
**Designed primers:** for expression analysis of distal *vs.* proximal 3´UTR extensions			
**Gene symbol**	**Gene name**	**Assay coverage**	**Assay sequence**
Tfrc	Transferin receptor	Gene body, last exon	fw: ggaagttttgcacttatggtcag<br>rv: gacatttaacttggccacaactc
Grin2a	Glutamate receptor, ionotropic, NMDA2A (epsilon 1)	Distal 3´UTR	fw: tatctactcccaaatgaggacca<br>rv: agtccatctttaacccagtgtga
Grin2a	Glutamate receptor, ionotropic, NMDA2A (epsilon 1)	Proximal 3´UTR	fw: cacatgtcaccacattaacgact<br>rv: gtaagtttgcattcctcccttct
Grm5	Glutamate metabotropic receptor 5	Distal 3´UTR	fw: atcctgtgctagtctcccag<br>rv: ccaggcctcataccctcttc
Grm5	Glutamate metabotropic receptor 5	Exon 9/11	fw: gccaactttaacgaggccaa<br>rv: cgcacatttctctccggttt
**Designed primers:** for Gadd45α pulldown experiments			
**Gene symbol**	**Gene name**	**Assay coverage**	**Assay sequence**
Sdha	Succinate Dehydrogenase Complex Flavoprotein Subunit A	3´UTR	fw: tctggactgtataagagcaagctg<br>rv: cattcacagtgagaagtagacaatga
Map2k6	Mitogen-Activated Protein Kinase 6	3´UTR	fw: ggtgggtttacagggtgaag<br>rv: cagggttcaatgacctcaaga
Grin2a	Glutamate receptor, ionotropic, NMDA2A (epsilon 1)	3´UTR	fw: tcaaagagccatgcctgaat<br>rv: cagaactgacctggctctca
Grm5	Glutamate metabotropic receptor 5	Exon 11/11	fw: atgacgaccttcgcagagat<br>rv: ctgagctcctccggtcac
Grin2b	Glutamate receptor, ionotropic, NMDA2B (epsilon 2)	3´UTR	fw: ctctgatatcaatgcccaagg<br>rv: gtgatgcctgggatacagaac
Kcnq3	Potassium voltage-gated channel, subfamily Q, member 3	3´UTR	fw: aagtgaaggaaggggttgct<br>rv: cagacaggaagaaatatgggtca
**Designed primers:** for *Gadd45a*^{K45E} point mutation and sequencing validation			
**Gene symbol**	**Gene name**	**Assay coverage**	**Assay sequence**
Gadd45a (point mutation)	Growth arrest and DNA-damage inducible protein 45 alpha	Region surrounding codon 45	fw: ctacgttgagcagctcggcagcctcgtacac<br>rv: gtgtacgaggctgccgagctgctcaacgtag
Gadd45a (sequencing)	Growth arrest and DNA-damage inducible protein 45 alpha	WPRE	rv: catagcgtaaaaggagcaaca

Appendix Table S2. List of primers used in qPCR and/or mutagenesis studies with the region covered and the sequence or the code for the Taqman assays.
