# Supplementary material for: Gadd45α modulates aversive learning through post‐transcriptional regulation of memory‐related mRNAs
Source: EMBO Rep. 2019 Apr 4;20(6):e46022. doi: 10.15252/embr.201846022 (PMC6549022; doi:10.15252/embr.201846022)
Supplement: Supplementary file 2 — Expanded View Figures PDF [file EMBR-20-e46022-s002.pdf]

Expanded View Figures

Figure EV1. Basic behaviors were unaffected in *Gadd45a*-KO mice (n = 12–16) as compared to *Gadd45a*-WT mice (n = 11–12).

A No genotype differences were found in the holeboard test in terms of locomotion (total ambulation). Similarly, general exploration (% of internal ambulation and head dipping frequency) was equal between genotypes. Values shown are mean ± SEM; unpaired t-test.

B The elevated plus-maze showed no significant differences in terms of locomotion (total entries), but a tendency toward increased anxiety (reduced % of open arm entries and time) in *Gadd45a*-KO mice. Values shown are mean ± SEM; unpaired t-test.

C The light/dark test corroborated the absence of significant genotype differences in anxiety-like behavior, measuring transitions between compartments, time in lit compartment, and latency to first visit the lit compartment. Values shown are mean ± SEM; unpaired t-test.

D Differences in depressive-like behavior (characterized by a decrease in swimming and increase in floating) were not found between genotypes. Values shown are mean ± SEM; unpaired t-test.

E *Gadd45a*-KO mice displayed normal recognition learning. Discrimination index (right panel) was significantly reduced in both genotypes during the retrieval sessions (1 and 24 h after object exposure), indicating the recognition of the familiar object. Total exploration (left) of both objects was reduced as a result of task habituation. Values shown are mean ± SEM; two-way ANOVA and Bonferroni post hoc test: *$P < 0.05$, **$P < 0.01$.

F *Gadd45a*-KO mice showed a reduced spatial navigation capability. Schematic representation of the water cross-maze setup (left panel). Learning curves of *Gadd45a*-WT and *Gadd45a*-KO mice (right panel) were similar during the acquisition phase (sessions 1 and 2), but differed significantly during the consolidation phase (sessions 2–3, and sessions 4–5). Values shown are mean ± SEM; Bonferroni post hoc test showing significant differences between sessions in both genotypes: ##$P < 0.01$ or t-test showing significant differences between genotypes for the sessions indicated: *$P < 0.05$, **$P < 0.01$.

G *Gadd45a*-KO mice showed a decreased amygdala-dependent fear memory. Left panel shows % of freezing of *Gadd45a*-WT and their *Gadd45a*-KO littermates during the 200-s tone presented in the extinction sessions plotted in 20-s time bins. Total percentage of freezing for each extinction session is depicted on the right panel. Note that the absence of Gadd45α leads to a consistent reduction in memory retention. Values shown are mean ± SEM; two-way ANOVA and Bonferroni post hoc test: *$P < 0.05$, **$P < 0.01$, ***$P < 0.001$.

H mRNA levels of *Gadd45* gene family and memory-related genes in hippocampal wild-type tissue (n = 6) measured by qPCR. Compared to *Gadd45a*, *Gadd45b* (t_{10} = 3.521) and *Gadd45g* (t_{10} = 4.601) showed significantly increased mRNA levels. *Gadd45a* mRNA levels are similar to other memory-related genes such as *Bdnf* and *Reelin*. Values shown are mean ± SEM; one-way ANOVA and unpaired t-test: **$P < 0.01$, ***$P < 0.001$.

 © 2019 The Authors

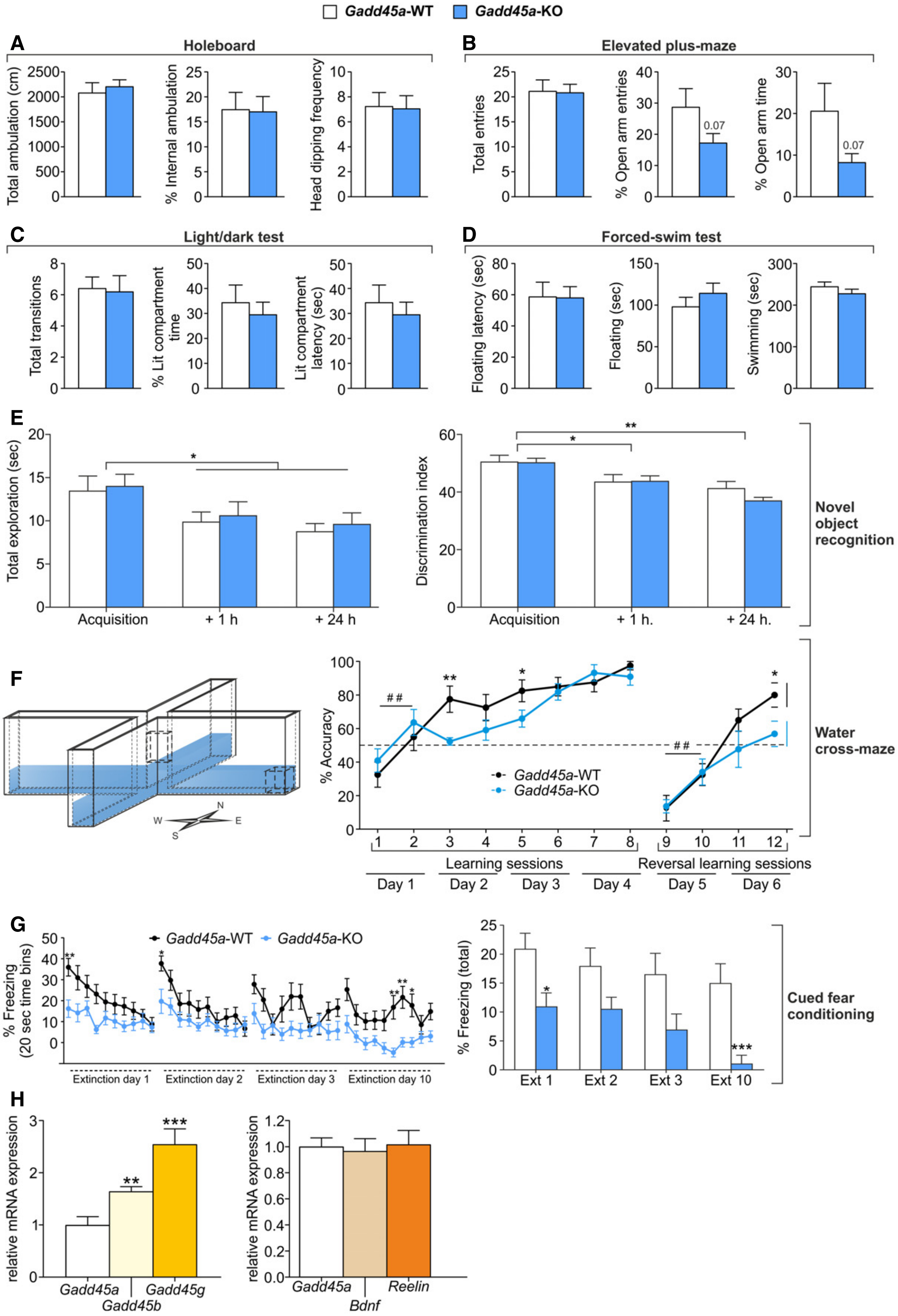

Gadd45a-WT
Gadd45a-KO
A
Holeboard
Total ambulation (cm)
% Internal ambulation
Head dipping frequency
B
Elevated plus-maze
Total entries
% Open arm entries
% Open arm time
0.07
C
Light/dark test
Total transitions
% Lit compartment time
Lit compartment latency (sec)
D
Forced-swim test
Floating latency (sec)
Floating (sec)
Swimming (sec)
E
Total exploration (sec)
Discrimination index
Acquisition
+ 1 h
+ 24 h
Novel object recognition
F
% Accuracy
Learning sessions
Reversal learning sessions
Day 1
Day 2
Day 3
Day 4
Day 5
Day 6
Water cross-maze
G
% Freezing (20 sec time bins)
Extinction day 1
Extinction day 2
Extinction day 3
Extinction day 10
% Freezing (total)
Ext 1
Ext 2
Ext 3
Ext 10
Cued fear conditioning
H
relative mRNA expression
Gadd45a
Gadd45b
Gadd45g
Bdnf
Reelin

Figure EV1.

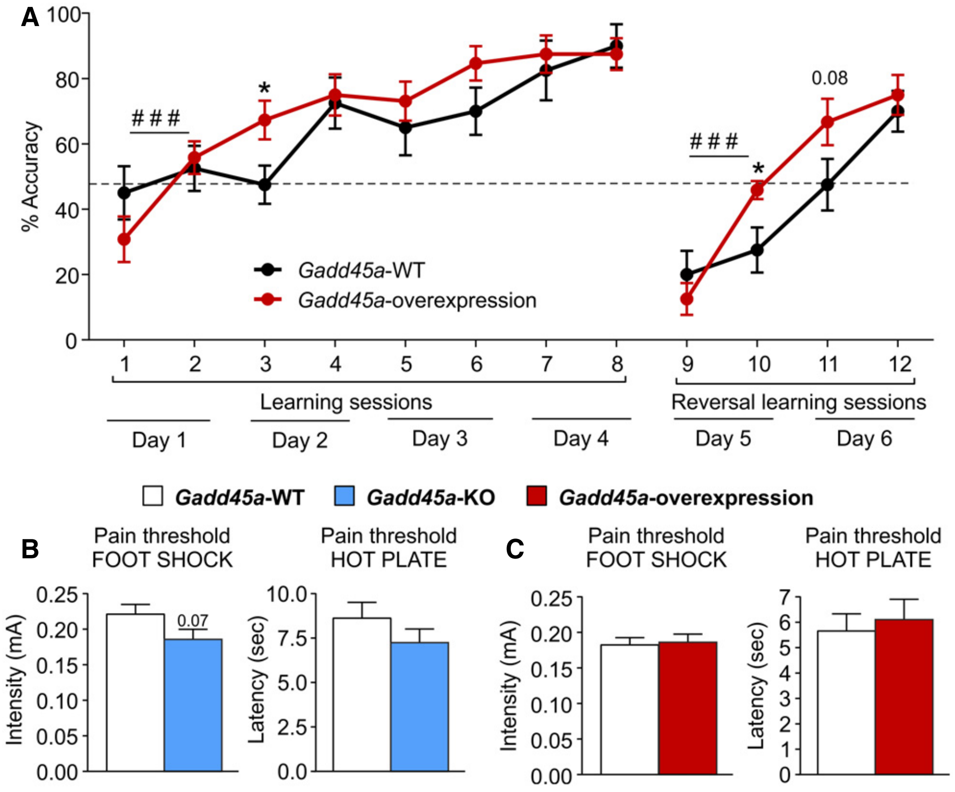

Figure EV2. *Gadd45a* overexpression enhanced memory consolidation without affecting pain sensitivity.

A *Gadd45a*-overexpressing mice (n = 12) have an increased spatial navigation based on a constantly higher % of accuracy to locate the platform during the water cross-maze, as compared to their *Gadd45a*-WT littermates (n = 10). Values shown are mean ± SEM; Bonferroni post hoc test showing significant differences between sessions in *Gadd45a*-overexpressing mice: $^{###}P < 0.001$ or t-test showing significant differences between genotypes for the sessions indicated: $^{*}P < 0.05$.

B Minimum intensity at which mouse reacted to a foot shock (left panel) and latency to the first sign of discomfort in the hot plate test (right panel) were not significantly different between *Gadd45a*-WT (n = 11, open bars) and *Gadd45a*-KO mice (n = 11, blue bars). Values shown are mean ± SEM; unpaired t-test.

C *Gadd45a*-overexpressing mice (n = 12, red bars) showed very similar pain thresholds in foot shock and hot plate tests as compared to their *Gadd45a*-WT littermates (n = 10, open bars). Values shown are mean ± SEM; unpaired t-test.

 © 2019 The Authors

Figure EV3. Analyses of synaptic transmission and plasticity.

A Paired-pulse ratio before (triangles) and after (circles) LTP induction in hippocampi from *Gadd45a*-WT (white symbols, n = 14) and *Gadd45a*-KO (blue symbols, n = 11) at different stimulation intervals. Note that LTP did not change the paired-pulse ratio, indicating that LTP was predominantly mediated by post-synaptic mechanisms. Values shown are mean ± SEM.

B Synaptic fatigue during the first stimulus train was similar in both genotypes, demonstrating that differences in LTP were not caused by differences in synaptic responsiveness to stimulation (n = 13 for *Gadd45a*-WT, n = 11 for *Gadd45a*-KO). Values shown are mean ± SEM.

C The paired-pulse ratio (before LTP induction: triangles, after LTP induction: circles) did not differ between *Gadd45a*-overexpressing mice (red symbols, n = 11) and their corresponding *Gadd45a*-WT littermates (white symbols, n = 10). Values shown are mean ± SEM.

D Synaptic fatigue during the first stimulus train was similar in both genotypes, demonstrating that differences in LTP were not caused by differences in synaptic responsiveness to stimulation (n = 7 for *Gadd45a*-WT, n = 10 for *Gadd45a*-overexpression). Values shown are mean ± SEM.

E Localization of the stimulating (cortical inputs of the amygdala) and recording (lateral nucleus of the amygdala) electrodes for LTP experiments in the amygdala. Scale bars: 500 μm (black bar).

F Slope of field potential responses in the lateral nucleus of the amygdala from *Gadd45a*-WT (n = 11 slices) and *Gadd45a*-KO mice (n = 8 slices) during LTP induction. Note stable expression of LTP in both genotypes but significantly ($P < 0.05$, Student's t-test) decreased LTP in *Gadd45a*-KO. Values shown are mean ± SEM.

G As in the hippocampus, paired-pulse ratio before (triangle) and after (circles) LTP induction did not differ between *Gadd45a*-WT (white symbols, n = 11) and *Gadd45a*-KO mice (blue symbols, n = 8). Values shown are mean ± SEM.

H Synaptic fatigue was not different in *Gadd45a*-overexpressing mice (blue bars) and controls (white bars), indicating that differences in LTP were not caused by differences in synaptic responsiveness to stimulation. Values shown are mean ± SEM.

© 2019 The Authors

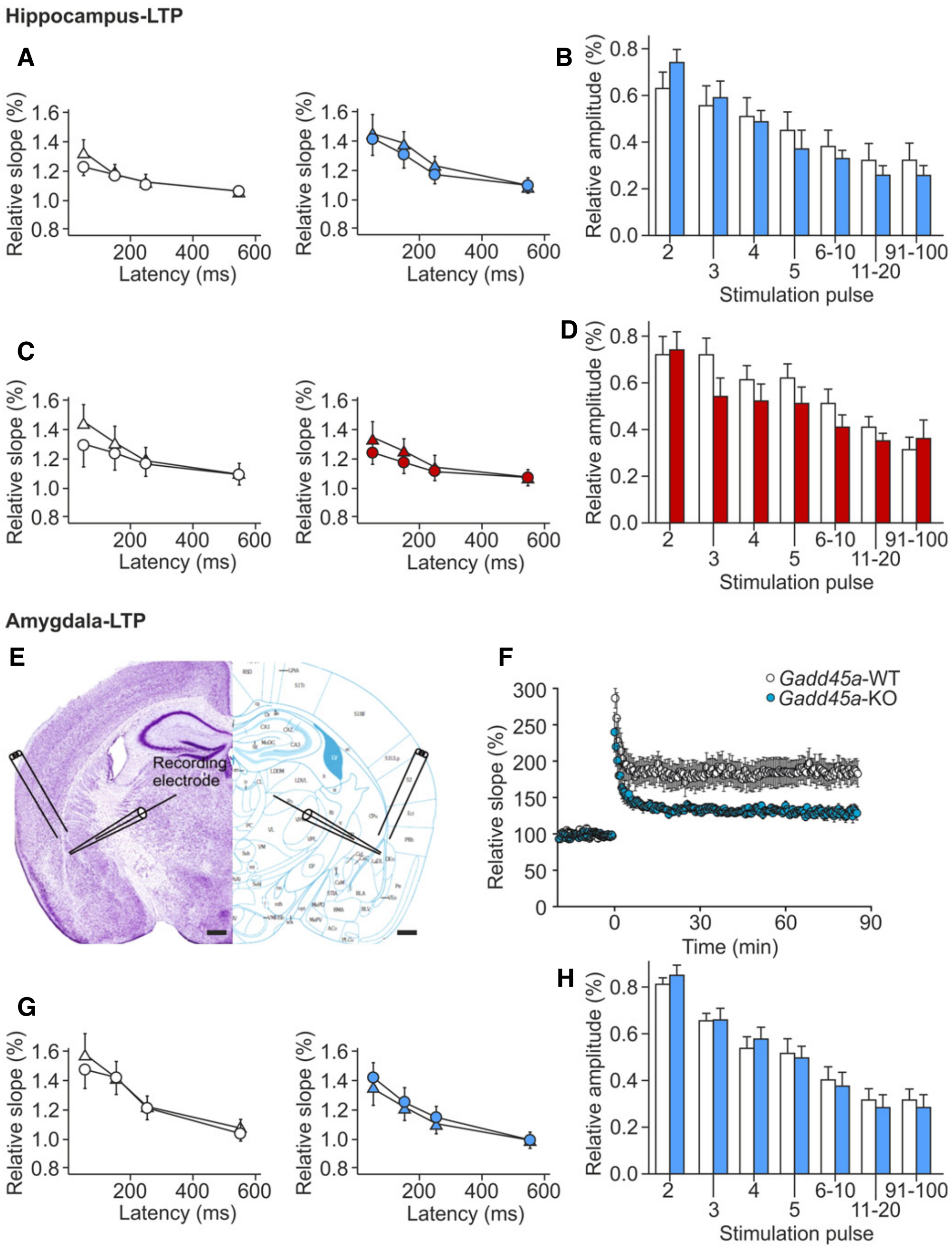

Figure EV3.

© 2019 The Authors

Figure EV4. Complementary analysis of Gadd45α-dependent post-transcriptional regulation of memory-related genes.

A, B Read coverage profiles (*y*-axis = reads per million reads) of representative *Gadd45a*-WT (black) and *Gadd45a*-KO (blue) samples for *Map2K6* (A), and a detailed view of its 3′UTR (B). Note that *Map2K6* contains an extended 3′UTR, but is not under Gadd45α-dependent regulation of 3′UTR stability. Additionally, the absence of a 5′<3′ gradient despite the presence of a long unannotated 3′UTR suggests gene specificity in the action of Gadd45α.

C Normalized RNA-seq read coverage of the annotated exons and introns of the indicated genes. Note that while exonic coverage is strongly reduced in *Gadd45a*-KO mice, intronic coverage is not or is only mildly affected (for *Grm5*), indicating that Gadd45α mediates post-transcriptional, rather than transcriptional regulation ($n = 6$ for all groups). Values shown are mean ± SEM; Student's *t*-test: $^{**}P < 0.01$.

D Pre-mRNA analysis of *Grin2a* (upper panel) and *Grm5* (lower panel) by intronic qPCR of *Gadd45a*-WT (control: no PA, $n = 6$; PA + 1 h, $n = 6$) and *Gadd45a*-KO hippocampal samples (control: no PA, $n = 6$; PA + 1 h, $n = 6$). Note that pre-mRNA levels for both transcripts are very similar in all experimental groups, corroborating the absence of transcriptional effects. Values shown are mean ± SEM; two-way ANOVA and Bonferroni post hoc test.

E qPCR analysis of *Grin2a* (left) and *Grm5* (right) mRNA levels of *Gadd45a*-WT (control: no PA, $n = 6$; PA + 1 h, $n = 6$) and *Gadd45a*-overexpressing mice (control: no PA, $n = 6$; PA + 1 h, $n = 6$). Note that when using random-primed cDNA, a significant increase only appeared for *Grm5* (genotype effect, $F_{1,20} = 6.637$). Values shown are mean ± SEM; two-way ANOVA and Bonferroni post hoc test: $^{*}P < 0.05$.

F Same qPCR analysis as in (E) but using oligo(dT)-primed cDNA. Note in this case, significant differences appeared for both *Grin2a* (genotype effect, $F_{1,20} = 5.031$) and *Grm5* (genotype effect, $F_{1,20} = 12.94$). Values shown are mean ± SEM; two-way ANOVA and Bonferroni post hoc test: $^{*}P < 0.05$, $^{**}P < 0.01$.

© 2019 The Authors

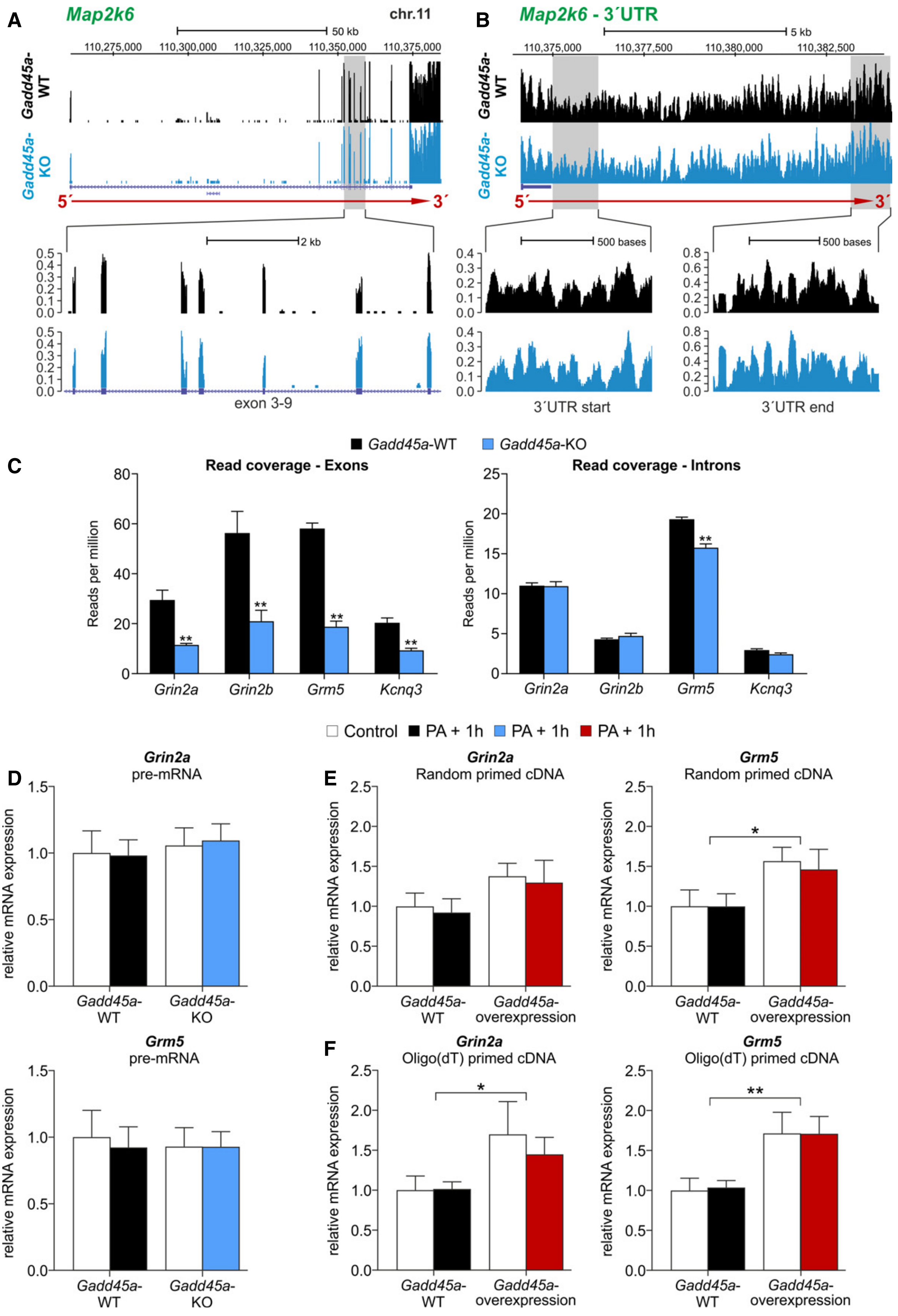

Figure EV4.

 © 2019 The Authors

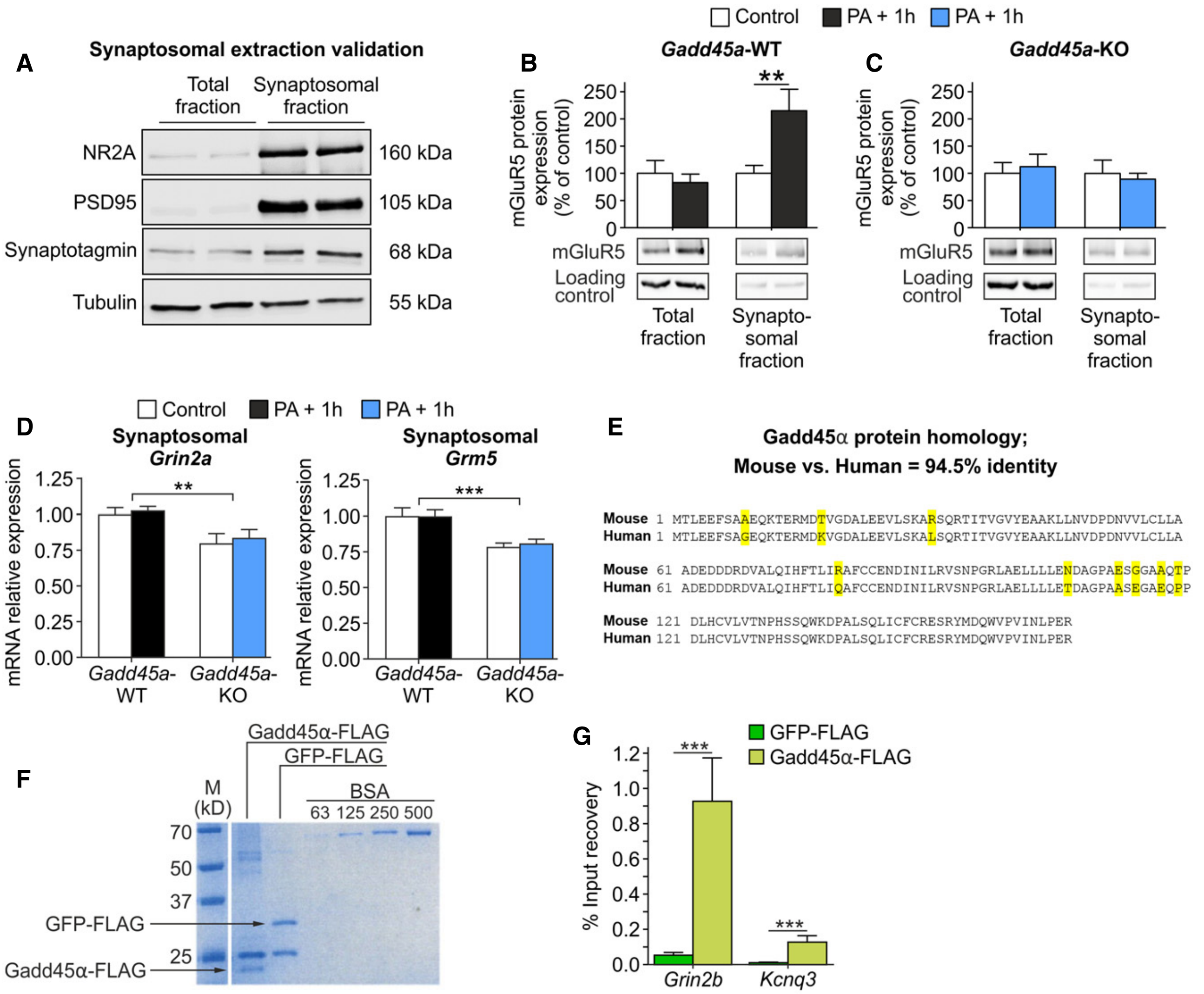

Figure EV5. Complementary data on the effect of Gadd45α on synaptosomal composition and its mRNA interaction.

A Representative Western blot analysis showing two wild-type C57BL/6N hippocampal total and synaptosomal protein fractions, respectively. Proteins known to be expressed in membranes of synaptosomes (NR2A, PSD95, synaptotagmin) were enriched in synaptosomal fraction as compared to total fraction. Tubulin (55 kDa) and synaptotagmin (68 kDa) served as loading controls for the total and synaptosomal fractions, respectively.

B, C mGluR5 protein levels (150 kDa) were increased in synaptosomal fractions of *Gadd45a*-WT hippocampi (B) 1 h after PA exposure (t_{19} = 3.121). (C) In *Gadd45a*-KO, mGluR5 levels were not altered during memory formation. The number of samples analyzed is the same as in Fig 5A and B. In control groups, 27 samples were analyzed (*Gadd45a*-WT, n = 13; *Gadd45a*-KO, n = 14), and in PA-exposed groups, 28 samples were analyzed (*Gadd45a*-WT = 14, *Gadd45a*-KO = 14). Values shown are mean ± SEM; two-way ANOVA and Bonferroni post hoc test: $^{**}P < 0.01$.

D mRNA levels of *Grin2a* (left panel) and *Grm5* (right panel) in synaptosomal fractions of *Gadd45a*-WT (control, n = 6; PA + 1 h, n = 6) and *Gadd45a*-KO (control, n = 6; PA + 1 h, n = 6) hippocampal samples. Note that mRNA levels of both transcripts were significantly decreased in *Gadd45a*-KO samples (*Grin2a*, $F_{1,20}$ = 13.55; *Grm5*, $F_{1,20}$ = 23.9) under both control and PA + 1 h conditions. Values shown are mean ± SEM; two-way ANOVA and Bonferroni post hoc test: $^{**}P < 0.01$, $^{***}P < 0.001$.

E Sequence homology between mouse and human Gadd45α protein shows 94.5% identity with nine residue mismatches (yellow).

F Coomassie gel of purified Gadd45α-FLAG and GFP-FLAG, and increasing concentrations of BSA, which served as reference to estimate the amount of purified Gadd45α-FLAG and GFP-FLAG used for the pulldown experiments.

G Percentage of input recovery was significantly enhanced in the Gadd45α-FLAG-bound fraction (light green bars) for *Grin2b* (t_{22} = 4.992) and *Kcnq3* (t_{22} = 3.267) mRNA as compared to GFP-FLAG-bound fraction (dark green bars) (n = 12 for all groups). Values shown are mean ± SEM; two-way ANOVA and Bonferroni post hoc test: $^{***}P < 0.001$.

Source data are available online for this figure.
