# Supplementary figures and images for: Gadd45α modulates aversive learning through post‐transcriptional regulation of memory‐related mRNAs
Source: EMBO Rep. 2019 Apr 4;20(6):e46022. doi: 10.15252/embr.201846022 (PMC6549022; doi:10.15252/embr.201846022)

Figure EV5B and C – Source data   = Samples displayed in main figures

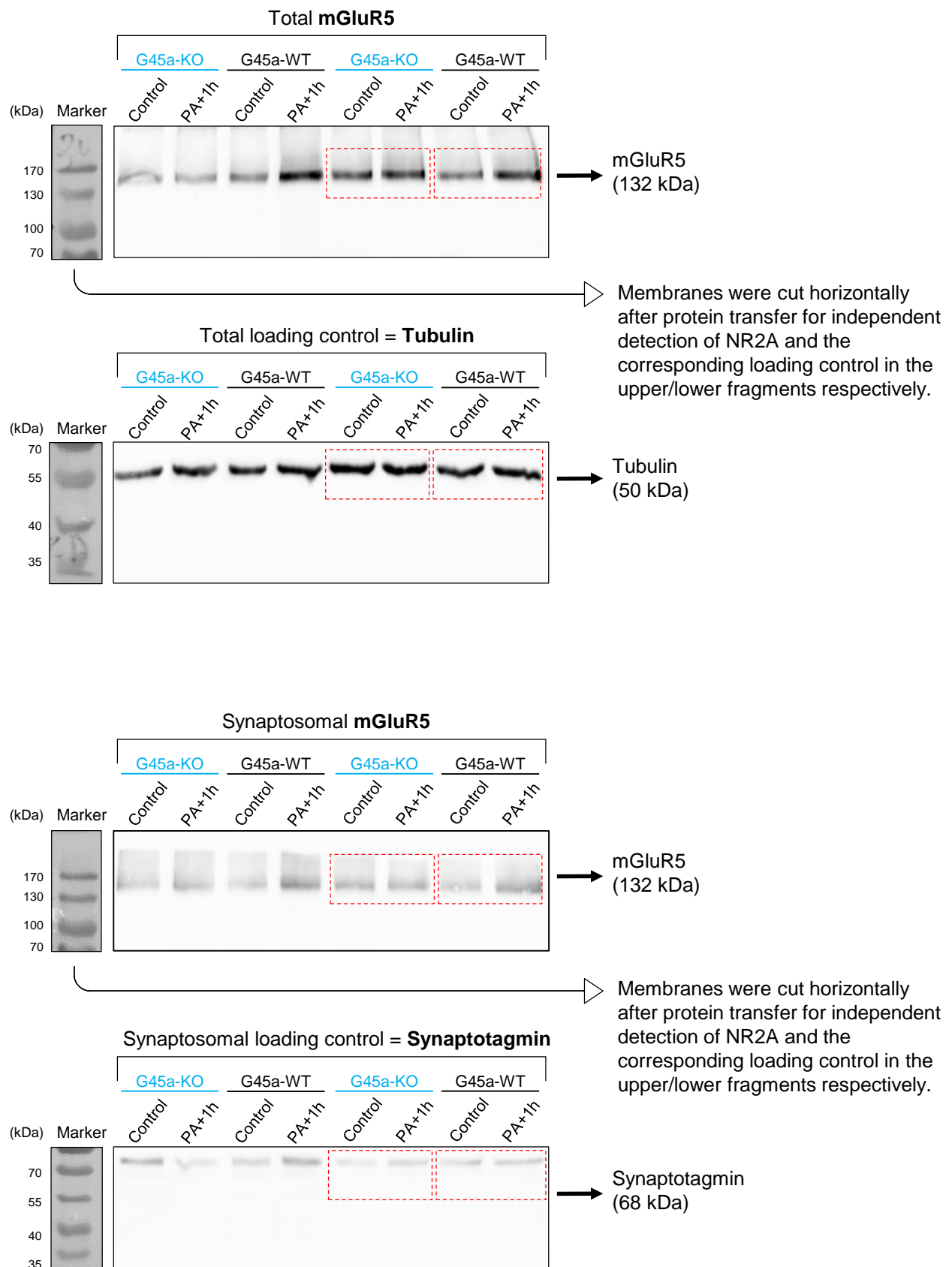

Supplement: Supplementary file 5 — Source Data for Expanded View [file EMBR-20-e46022-s008.zip › embr201846022-sup-0008-SDataFigEV5B_and_C.pdf]

**Figure 2D – Source data**

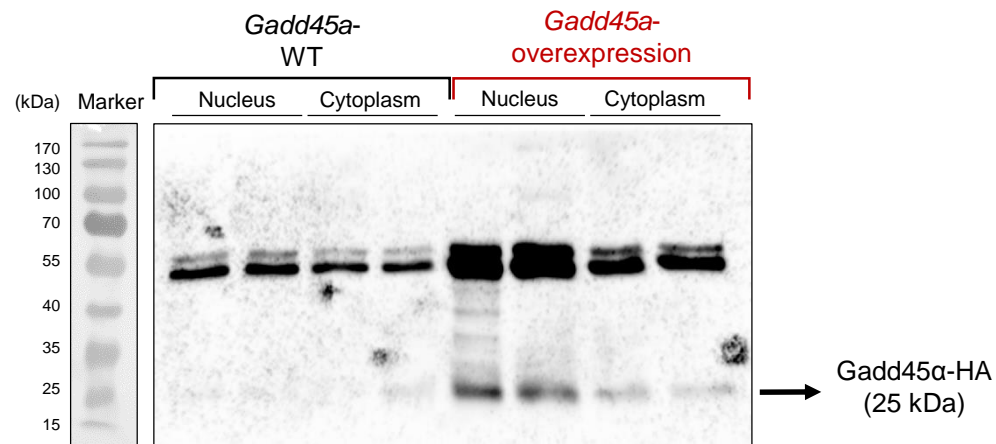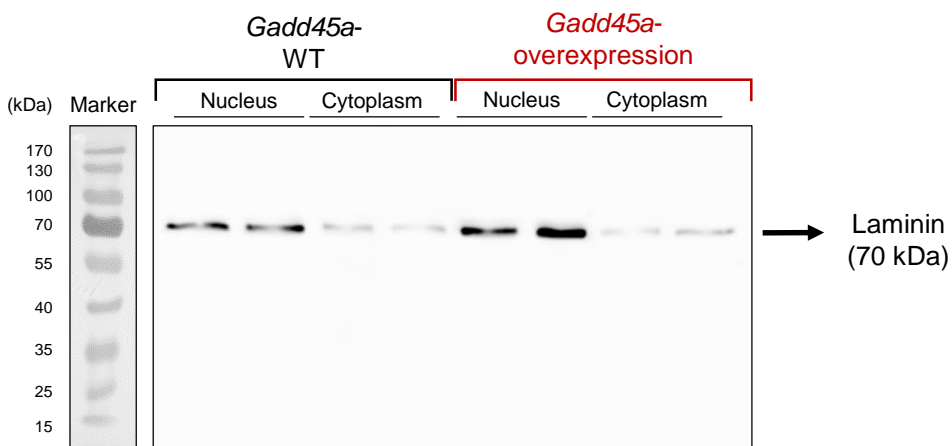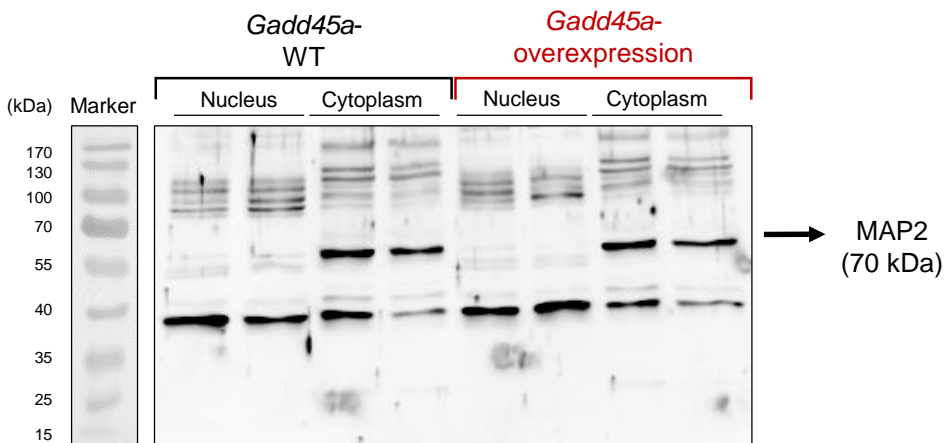

Supplement: Supplementary file 7 — Source Data for Figure 2 [file EMBR-20-e46022-s005.pdf]

Figure 5A and B – Source data

  = Samples displayed in main figures

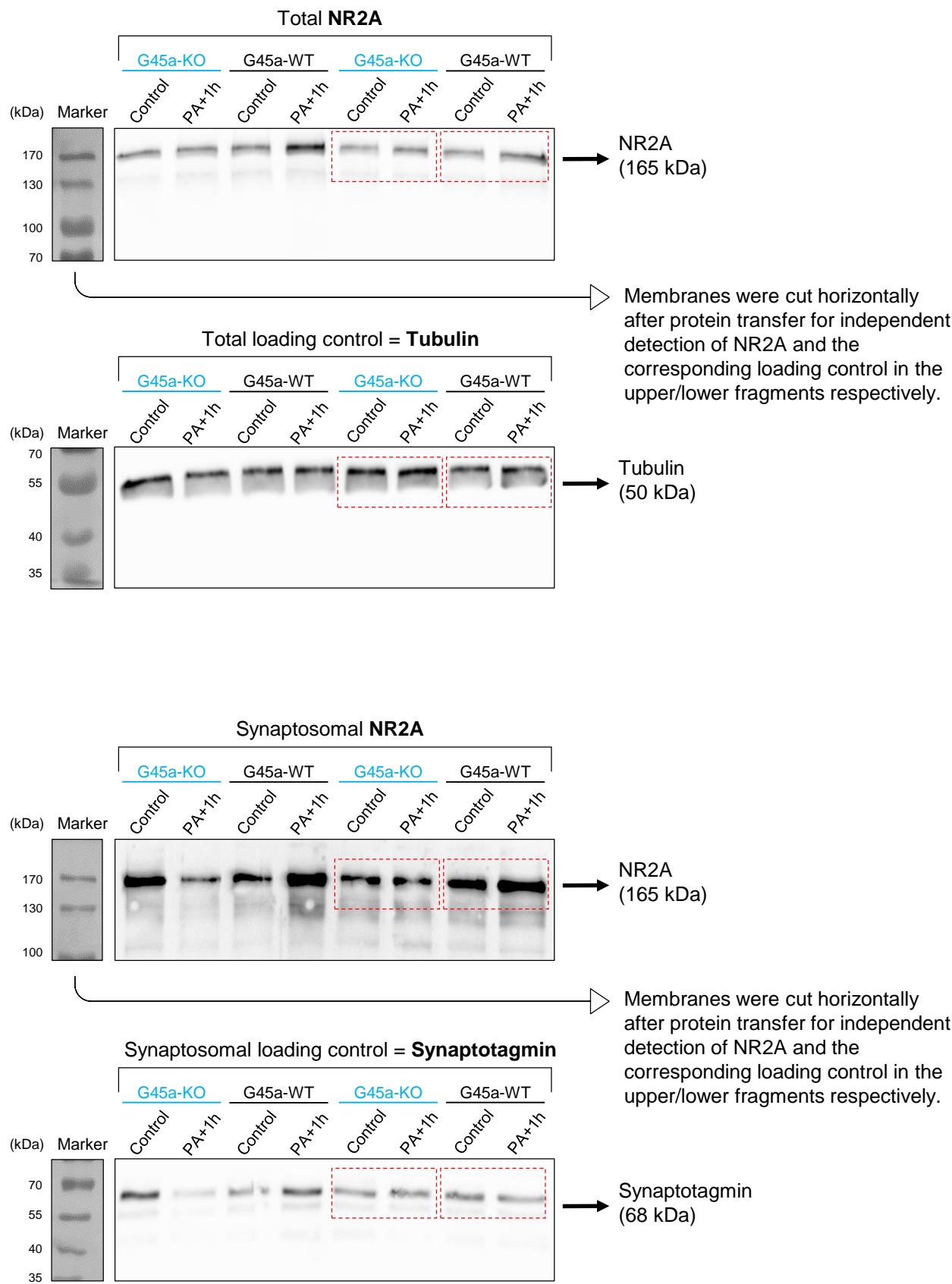

Supplement: Supplementary file 8 — Source Data for Figure 5 [file EMBR-20-e46022-s006.pdf]

Figure 6C – Source data

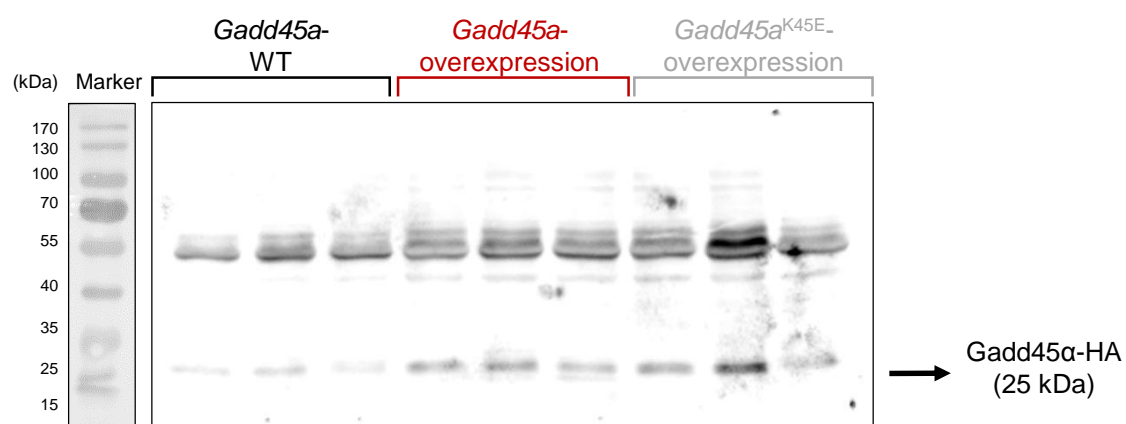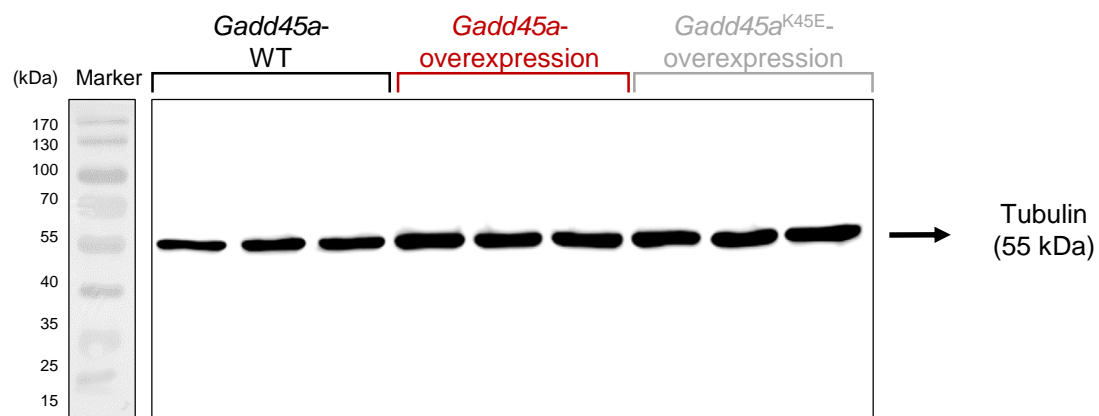

Supplement: Supplementary file 9 — Source Data for Figure 6 [file EMBR-20-e46022-s007.pdf]
